# Preliminary clinical and radiographic outcomes of proximal humeral fractures: comparison of ALPS and PHILOS plating in Asian patients in Taiwan

**DOI:** 10.1186/s13018-020-01846-9

**Published:** 2020-08-28

**Authors:** Chun-Yen Chen, Hao-Wei Chang, Shang-Lin Hsieh, Chien-Chun Chang, Chun-Hao Tsai, Yi-Wen Chen, Tsung-Li Lin, Chin-Jung Hsu

**Affiliations:** 1 Department of Orthopedics, China Medical University Hospital, Taichung, Taiwan; 2 Ph.D. Degree Program of Biomedical Science and Engineering, National Chiao Tung University, Hsinchu, Taiwan; 3 Department of Sports Medicine, China Medical University, Taichung, Taiwan; 4 Graduate Institute of Biomedical Sciences, China Medical University, Taichung, Taiwan; 5 3D Printing Medical Research Institute, Asia University, Taichung, Taiwan; 6 School of Chinese Medicine, China Medical University, Taichung, Taiwan

**Keywords:** Proximal humerus, ALPS locking plate, PHILOS locking plate, Fracture fixation, Asian patient, Treatment outcome, Complication

## Abstract

**Background:**

Up to 20% of proximal humeral fractures need to be treated operatively. However, numerus complications were reported by using fixed angled locking plates. The ALPS Proximal Humerus Plating System is a new design implant with novel design features.

The aim of this study was to compare the preliminary clinical outcomes and complications of proximal humeral fractures treated with either ALPS or the proximal humeral internal locking system (PHILOS) in Asian patients in Taiwan.

**Methods:**

Between January 2016 and December 2018, 66 patients with displaced proximal humeral fractures were analyzed retrospectively, of whom 31 underwent ALPS implant treatment and 35 underwent PHILOS implant treatment. Intraoperative blood loss and operation time, postoperative Constant-Murley Shoulder Outcome (Constant-Murley) score, and complications variables were recorded for the comparison. All cases were regularly followed up for at least 1 year.

**Results:**

The mean follow-up period was 400.8 days (range, 367–446 days). Union was achieved in 98.5% of patients (65/66). The ALPS group yielded similar radiologic and clinical outcomes to the PHILOS plating group for treating displaced proximal humeral fractures, including operation time, intraoperative blood loss, the Constant-Murley score, and varus malunion (*P* > 0.05, respectively). However, the incidence of total postoperative complications in the ALPS group was significantly lower than in the PHILOS group (*P* < 0.05). There was a trend of a lower complication rate of screws/pegs protrusion, avascular necrosis, subacromial impingement, postoperative infection, and reoperation in the ALPS group, although it was not statistically significant (*P* > 0.05, respectively).

**Conclusion:**

The ALPS group yielded similar radiologic and clinical outcomes to the PHILOS plating group for displaced proximal humeral fractures, but the ALPS group had a significantly lower total rate of complications. Therefore, ALPS may be a better option for treating proximal humeral fractures. Further larger clinical studies are needed to confirm the findings presented here.

**Trial registration:**

Retrospective study

## Background

Fracture of the proximal humerus is the seventh most common cause of fracture in adults [1] and the second most common cause of upper extremity fractures, accounting for approximately 5% of all fractures [2]. From 1990 to 2020, the age-adjusted incidence of this fracture type has increased by 15% annually, most likely because of the increasing prevalence of osteoporotic injuries and the mean age of the affected patients [3].

Although most proximal humeral fractures are treated nonoperatively, certain complex fractures require surgical treatment [4]. Surgical treatment includes use of various techniques such as intramedullary locking nail osteosynthesis, open reduction and locking plate osteosynthesis, primary hemiarthroplasty, and reverse shoulder arthroplasty [5]. Numerous surveys have suggested that angular stable implants provide sufficient fracture stabilization in older patients [6, 7]. Of these implants, the proximal humeral internal locking system (PHILOS) (Synthes, Solothurn Switzerland) is a well-established and widely reported implant with good results [8, 9]. However, it is frequently associated with high complication rates (up to 28.2%), such as humeral head avascular necrosis (AVN), secondary fracture displacement, screw cut-out with intraarticular penetration, or subacromial impingement with plate [10–12]. Another type of osteosynthesis, the ALPS Proximal Humerus Plating System (Zimmer Biomet, Warsaw, Indiana, USA), has novel design features, such as smooth blunt-ended pegs, multi-directional medial calcar locking screws, increased numbers of suture holes, and a lower position of the plate, to reduce the risk of the abovementioned complications [13]. However, few studies have addressed the outcome of this novel locking plate system or have compared ALPS and PHILOS for the fixation of proximal humeral fractures. In this study, we report the preliminary clinical and radiographic outcomes of ALPS treatment for proximal humeral fractures compared to PHILOS treatment in an Asian population in Taiwan.

## Methods

**Fig. 1:**
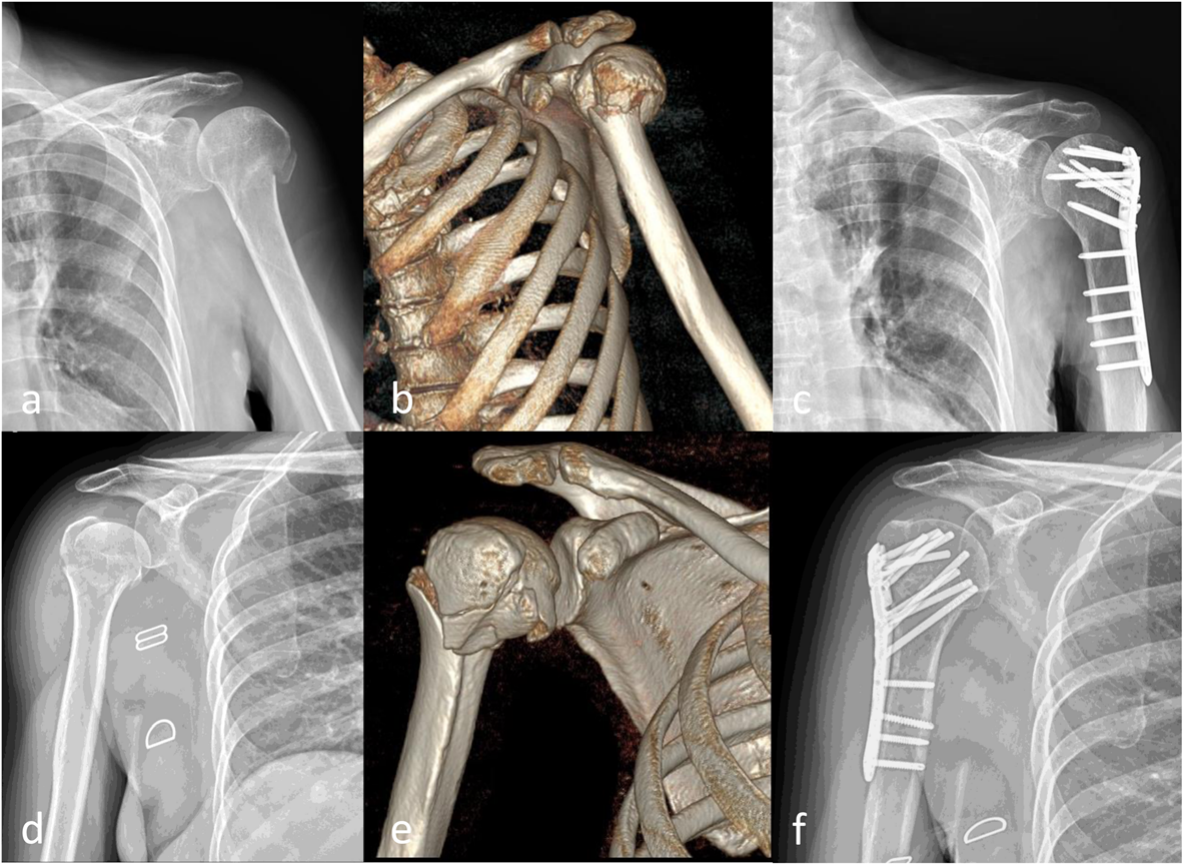
**a** Radiograph of a 79-year-old woman with Neer classification of three-part proximal humeral fracture. **b** Three-dimensional (3D) computed tomography (CT) reconstruction. **c** Radiograph at final follow-up 360 days after surgery with PHILOS plating. **d** Radiograph of a 72-year-old woman with Neer classification of three-part proximal humeral fracture. **e** CT scan with 3D reconstruction. **f** Radiograph at final follow-up 375 days after surgery with ALPS plating

This was a non-randomized retrospective study conducted between January 2016 and December 2018. The study was approved by the local IRB/Research Ethics Committee (approval numberCMUH102-REC2-062). A total of 66 patients with proximal humeral fractures received an open reduction and internal fixation (ORIF) using locking plates. The inclusion criteria were presence of closed proximal humeral fractures (2-, 3-, 4-part, according to the Neer classification system [14]) and age > 20 years. Patients with glenohumeral joint dislocation, open, pathological, multiple fractures, or a follow-up period less than 1 year were excluded. Fractures were classified on the basis of preoperative plain radiographs with computed tomographic images. ORIF with ALPS or PHILOS was performed consecutively for these proximal humeral fractures. We used PHILOS in the early period and used ALPS consecutively in these fractures because ALPS was introduced after PHILOS in Taiwan. Thirty-five patients were treated with PHILOS (PHILOS group) (Fig. 1a–c), and 31 patients were treated with ALPS (ALPS group) (Fig. 1d–f).

**Fig. 2:**
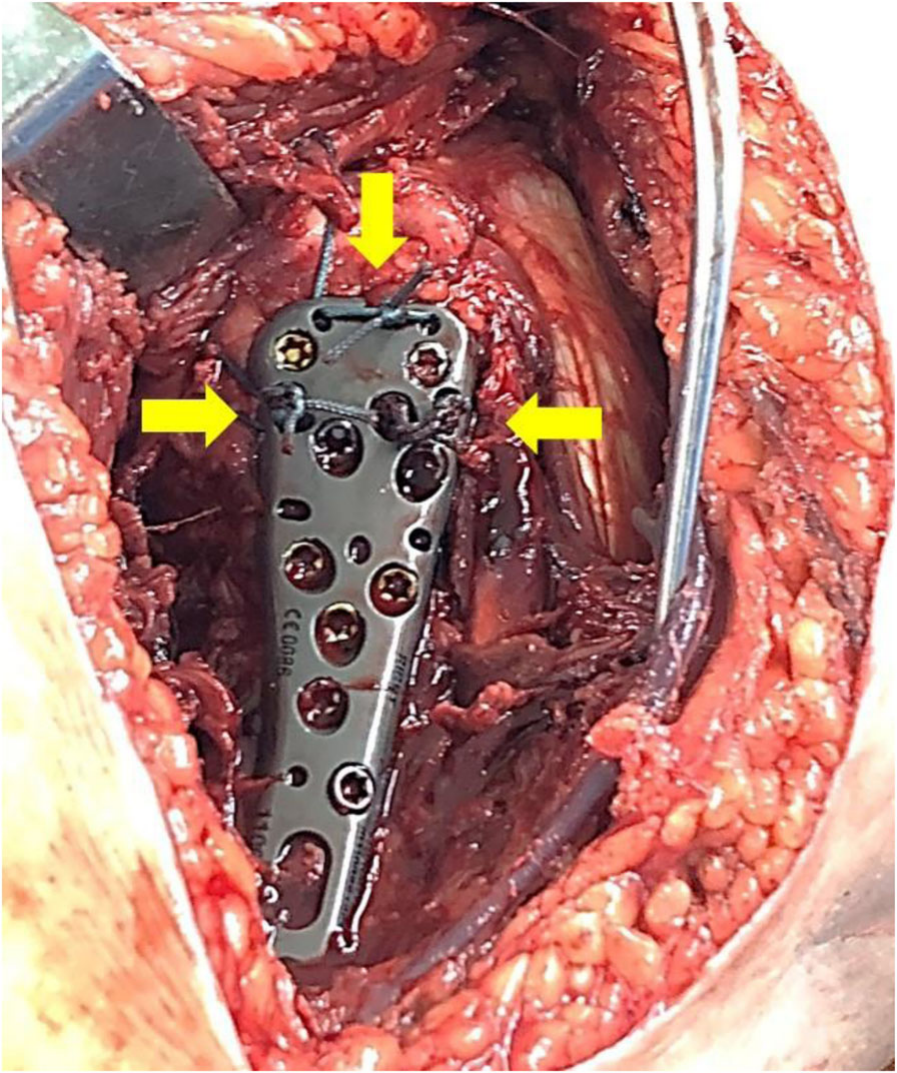
Three transosseous sutures at the GT fragment (yellow arrow) attached to the high-setting ALPS plate

The surgery in all cases was performed by two consultants specialized in upper limb surgery (Tsung-Li Lin and Chin-Jung Hsu) with patients under general anesthesia. The patients were placed in a supine position. The deltopectoral approach was applied [15]. First, the incision was made on the anterior aspect of the shoulder with exposure of the deltoid muscle. Second, the fracture was reduced, followed by the application of heavy sutures to control the rotator cuff and associated fragments. In the ALPS group, the high or low plate was chosen on the basis of the fracture pattern and to avoid impingement; proximal holes were drilled up to the subchondral bone and fixed using locking pegs rather than locking screws; then we chose the most suitable trajectory of the medial calcar screws or pegs under fluoroscopy. The remaining holes were fixed using locking screws. In both groups, the tuberosities were fixed with at least three transosseous sutures as augmentation and attached to the plate in all patients (Fig. 2).

Postoperatively, all patients underwent a similar physical therapy program. The shoulder was immobilized in an arm sling for the first 2 postoperative days. Gentle passive pendulum exercises were introduced after the suture removal to prevent shoulder stiffness. After 4 weeks, all patients began gentle passive flexion, abduction, and rotation exercises. Active exercise was prescribed after eight weeks. We reviewed patients’ charts including operation notes, preoperative or postoperative radiographic reports, and out-patient department records. The operation time, operation blood loss, bone union time, postoperative functional outcomes (Constant-Murley score), and complications were analyzed at the final follow-up. All results were compared between the two groups. The continuous variables were presented as mean ± standard deviation. The categorical variables are presented as number and percentage. The *t* test was used to compare the differences in the continuous variables, while the Fisher’s exact test was used for categorical variables between the two treatment groups. Statistical analyses were performed using SPSS for Windows version 24 (SPSS Inc., Armonk, NY). Statistical significance was set at *P* < 0.05.

## Results

The study cohort was comprised of 19 male and 47 female patients with a mean age of 58.9 years (range, 19–84 years). Thirty-six patients were older than 60 years and 30 patients were younger than 60 years. The traumatic mechanism for the fractures were simple falls in 28 patients and road traffic accidents in 38 patients. According to the Neer classification system, two-part fractures were noted in 19 patients, three-part fractures in 34 patients, and four-part fractures in 13 patients. The mean follow-up period was 400.8 days (range, 367–446 days). Of the 66 patients, 65 patients (98.5%, men, 18; women, 47) had complete bone union. Patients’ demographics and radiographic findings are shown in Table 1.

**Table 1:** Demographic data and radiographic findings of patients

	ALPS group	PHILOS group	*P* value
*N*	31	35	
Age in years (mean ± SD)	60.2 ± 12.6	56.1 ± 17.6	0.29
Gender (male:female)	06:25	13:22	0.11
Mechanism (high:low energy)	15:16	23:12	0.15
Fracture type (Neer II:III:IV parts)	07:17:07	12:17:06	0.56
Smoking	3	5	0.71
Follow-up (days)	405.1	397	0.81

*ALPS* ALPS Proximal Humerus Plating System, *PHILOS* proximal humeral internal locking system

In the ALPS group, the average surgery time was 137.0 min (range, 71–262 min), and the mean total intraoperative body blood loss was 229.6 cc (range, 10–800 cc). Two patients (6.5%, 2/31) underwent surgery using low plates. The mean Constant-Murley score was 76.4 (range, 37– 4). During the follow-up, varus malunion in two patients (6.5%, 2/31) was noted. In the PHILOS group, the average operation time was 135.7 min (range, 83–251 mins) and the amount of blood loss was 187.1 cc (range, 30–1150 cc). The postoperative mean Constant-Murley score was 73.2 (range, 32–95) at the last follow-up. Postoperative radiography on X-ray view revealed varus malunion in six patients (17.1%, 6/35). There were no statistically significant differences in clinical and operative findings between the two groups (*P* > 0.05) (Table 2).

**Table 2:** Radiologic, clinical, and operative results in both groups

	ALPS group	PHILOS group	*P* value
Operation time (min) (mean ± SD)	137.0 ± 45.3	135.7 ± 34.5	0.89
Intraoperative blood loss (mL)	229.7 ± 171.1	187.1 ± 192.9	0.35
Constant-Murley score	76.4 ± 13.6	73.2 ± 15.2	0.37
Varus malunion *n* (%)	2 (6.5%)	6 (17.1%)	0.27

*ALPS* ALPS Proximal Humerus Plating System, *PHILOS* proximal humeral internal locking system

We recorded complications such as screw/peg protrusion, AVN, subacromial impingement, postoperative infection, shoulder stiffness, greater tuberosity (GT) loss reduction, or implant failure (Table 3).

**Table 3:** Postoperative complications in both groups

Complications	ALPS group *N* (%)	PHILOS group *N* (%)	*P* value
Total number	7 (22.6)	16 (45.7)	0.049
Screws/pegs protrusion	2 (6.5)	3 (8.6)	1
AVN	1 (3.2)	6 (17.1)	0.11
Subacromial impingement	0 (0)	4 (11.4)	0.12
Postoperative infection	0 (0)	1 (2.9)	1
Shoulder stiffness	2 (6.5)	2 (5.7)	1
GT loss reduction	2 (6.5)	0 (0)	0.22
Implant failure	0 (0)	0 (0)	1
Reoperation	1 (3.2)	2 (5.7)	1

*ALPS* ALPS Proximal Humerus Plating System, *AVN* avascular necrosis, *GT* greater tuberosity, *PHILOS* proximal humeral internal locking system

In comparison with the PHILOS group, the ALPS group had a significantly lower total complication rate (*P* = 0.049). There was a trend of lower complication rate of screws/pegs protrusion, AVN, subacromial impingement, and postoperative infection in the ALPS group. However, differences in the rate of specific complications between the two groups were not statistically significant. Shoulder stiffness was noted in two patients in both groups. We arranged a shoulder rehabilitation program to correct the stiffness at 2 months postoperatively. The patient population did not have any cases of implant failure. Reoperation was found in three patients (two patients in PHILOS group and one patient in ALPS group). In patients treated with PHILOS, the reoperations were related to a postoperative infection (*N* = 1) and a screw protrusion (*N* = 1). In the ALPS group, one patient with GT loss reduction accepted revision surgery and bone graft augmentation with success.

## Discussion

This was the first preliminary study to compare the clinical and radiographic outcomes of proximal humeral fractures treated with ALPS or PHILOS plating in Asian patients in Taiwan with at least 1 year of follow-up. Our preliminary results demonstrated that the ALPS group yielded similar radiologic and clinical outcomes to the PHILOS plating group for displaced proximal humeral fractures, including operation time, intraoperative blood loss, the Constant-Murley score, and varus malunion. However, the ALPS group resulted in a significantly lower total complication rate than the PHILOS group. There was also a trend of less complication rate of screws/pegs protrusion, AVN, subacromial impingement, postoperative infection, and reoperation in the ALPS group, although this was not statistically significant.

Operative treatment for comminuted and displaced proximal humeral fractures is complex and challenging. The angular locking plate has demonstrated good clinical outcomes [9, 14, 16–18]; however, it is frequently associated with high complication rates (up to 28.2%) [10–12]. The main complication associated with PHILOS plating is subacromial impingement. In an early series of 28 consecutive patients, up to 21.4% of patients experienced this complication, which resulted from the superior positioning of the PHILOS plate [6]. The authors suggested that the PHILOS plate should be placed more distally. The technique of using a K-wire inserted through a hole at the top of the plate and lined up with the tip of the GT was recommended with fluoroscopy. In contrast to PHILOS, ALPS with the design of a central K-wire hole keeps the plate in a proper position more easily. Moreover, ALPS can use two types of plate options: low- and high-sitting plates. A retrospective study revealed that none of the patients in whom low-sitting plates were used had subacromial impingement [19]. The current study demonstrated the decreased rate of subacromial impingement in the ALPS group (0/31, 0%), even though most patients (29/31, 93.5%) were treated with high-sitting plates, compared with four cases (4/35, 11.4%) in the PHILOS group.

The complication of screw/peg protrusions became bothersome due to the high prevalence of osteoporotic injuries and the mean age of the affected patients. Spross et al. reported that screw/peg protrusions occurred at a rate of 11.2% with PHILOS plating [17]. Clavert et al. described a similar higher rate of screw cut-out of up to 13.7% [12]. Moreover, a review study indicated that nearly half of the patients who experienced screw protrusions were older than 60 years, including when an angular stable locking plate was used [20]. Several studies on bone fixation using PHILOS have indicated that screw protrusion is the most common reason for revision surgery [17, 18]. In contrast to the locking screws construct, the ALPS novel design of smooth blunt-ended pegs not only decreased the symptoms of protrusion, but also theoretically griped the bone stock effectively. However, there have been no biomechanical or clinical studies to support the design of pegs in proximal humeral fractures. In the current study, the screw/peg protrusion rate was 6.5% (2/31) in the ALPS group and 8.6% (3/35) in the PHILOS group. All patients with this complication were older than 60 and suffered from collapse of the humeral head. There was no statistically significant difference in screw/peg protrusions between the two groups or the sub-group analysis of patients older than 60. One patient in the PHILOS group accepted revision surgery due to a screw protrusion. In the ALPS group, the two patients who suffered from peg protrusions were neither symptomatic nor had a of loss reduction. We noted that the novel design of the pegs in ALPS has a comparable effectiveness to grip the bone stock as in the PHILOS technique. Nevertheless, the use of blunt pegs prevents revision surgeries due to less irritation of the protrusion.

Although the sub-group analysis of the complication rate was not statistically significant, we observed AVN as the predominant complication in the PHILOS group.

This adverse outcome could be associated with shoulder pain, limited range of motion, and arthritis of the glenohumeral joint. Several observational studies have reported an AVN rate of 0 to > 30% in patients following PHILOS fixation [6, 10, 16, 18]. Boesmueller et al. revealed that the high rate of AVN could be attributed to the use of AO (Association of Osteosynthesis) and the Neer classification systems [21]. Cadaver studies have indicated that the posterior circumflex artery is the major contributing vessel to the humeral head [22]. However, an early report related to ALPS revealed a low AVN rate [19]. The ALPS peg fixation and central K-wire hole targeting design might have reduced the AVN rate by lowering vessel injuries and improving the humeral head bone stock reserve. In our study, there were six patients (6/35, 17.1%) who encountered AVN in the PHILOS group, which account for nearly half of the cases that developed complications. Conversely, we found that using ALPS may decrease the AVN rate (1/31, 3.2%). However, the decrease was not significant, and further investigation of AVN is needed due to the limited sample size used in the current study.

Medial calcar screws play a key role in locking plate fixation for proximal humeral fractures [10]. The oblique locking screw placed into the inferomedial aspect of the humeral head can counteract the varus deforming force and reduce the risk of subsequent varus collapse. A prior study showed that medial calcar screws should be positioned < 12 mm from the apex of the arch of the calcar or within the bottom 25% of the humeral head [23]. Due to the design of the fixed angle locking plate, the fixed medial calcar screw trajectory does not match the appropriate zone in populations with different bone sizes, especially in Asian patients. Numerus studies revealed higher varus malunion rates by using angular stable locking plates such as PHILOS plating, ranging from 16 to 54.3% [20, 24, 25]. Alternatively, ALPS has an advantage of implementing multi-directional medial calcar locking screws. This may allow surgeons to have greater freedom for positioning of the calcar screw in a precise position, irrespective of plate position or bone size. For the patients with medial comminutions, the ALPS novel design of the multi-directional medial calcar may help prevent varus malunion. In the current study, there were only two patients (6.5%, 2/31) who encountered varus malunion in the ALPS group compared to six patients (17.1%, 6/35) in the PHILOS group. However, further clinical studies are needed to clarify this point.

**Fig. 3:**
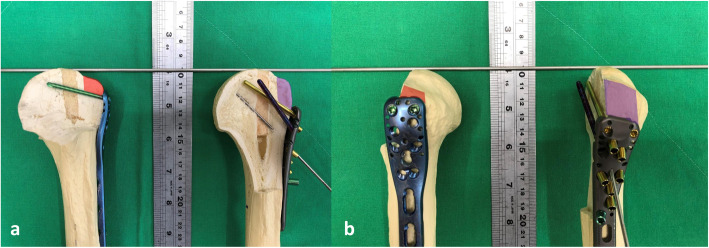
For sawbones of the same size, fewer GT fragments were covered by ALPS. (Red area indicates GT fragment without PHILOS coverage; purple area, GT fragment without ALPS coverage) **a** Anteroposterior view of plates. **b** Lateral view of plates. ALPS, Proximal Humerus Plating System; GT, greater tuberosity; PHILOS, proximal humeral internal locking system

Very few studies have focused on GT loss reduction of proximal humeral surgical treatments. Gillespie et al. reported a case series of 11 consecutive patients with isolated GT fractures [26], in which all patients experienced bone union without loss reduction after plate fixation. In the current study, GT loss reduction was noted in two patients in the ALPS group during the follow-up period. However, these two patients were treated with high-positioned plates, and one of the two received a further revision surgery with success. We applied PHILOS and ALPS on similarly sized sawbones to investigate the difference. The positions of PHILOS and ALPS were assessed according to the manufacturer. The results revealed that the distance between the GT and the apex of the plate was larger with ALPS than with PHILOS (Fig. 3). Although ALPS is designed to lower the complication rates of subacromial impingement, less buttressing coverage of the GT fragment was presented on the sawbone. Therefore, multiple transosseous sutures on the GT fragment should be considered for patients who are susceptible to fixation failure, such as those with low local bone mass density, increased age, and multifragmentary fracture patterns [27].

We acknowledge some limitations of this study. First, our study included a small sample size and was a retrospective study without randomization. Second, the follow-up period was relatively short in both groups; however, the average follow-up period was 1–2 years and did not justify forecasting of the long-term outcomes. Larger clinical studies are required to validate the findings presented here.

## Conclusions

The ALPS group yielded similar radiologic and clinical outcomes to the PHILOS plating group for displaced proximal humeral fractures, but the ALPS group had a significantly lower total rate of complications. Therefore, ALPS may be a more favorable treatment option than PHILOS for proximal humeral fractures. Further control based multi-center randomized control trials are required in the future to confirm the efficacy and potential advantages presented here.

## Data Availability

The dataset supporting the conclusions of this article is included within the article.

## Abbreviations

ALPS: ALPS Proximal Humerus Plating System
PHILOS: Proximal humeral internal locking system
AVN: Avascular necrosis
GT: Greater tuberosity
ORIF: Open reduction and internal fixation
AO: Association of Osteosynthesis
CT: Computed tomography
